# Supplementary material for: In situ or Ex situ Synthesis for Electrochemical Detection of Hydrogen Peroxide—An Evaluation of Co2SnO4/RGO Nanohybrids
Source: Micromachines (Basel). 2023 May 17;14(5):1059. doi: 10.3390/mi14051059 (PMC10221292; doi:10.3390/mi14051059)
Supplement: Supplementary file 1 [file micromachines-14-01059-s001.zip › micromachines-2356960-supplementary.pdf]

micromachines

# Supplementary information

*Article*

# *In situ* or *Ex situ* Synthesis for Electrochemical Detection of Hydrogen Peroxide—An Evaluation of $Co_2SnO_4$/RGO Nanohybrids

**Constanza J. Venegas [1], Fabiana A. Gutierrez [2,3], Nik Reeves-McLaren [4], Gustavo A. Rivas [5], Domingo Ruiz-León [6,*] and Soledad Bollo [7,8,*]**

1 Programa Institucional de Fomento a la Investigación, Desarrollo e Innovación, Universidad Tecnológica Metropolitana, Ignacio Valdivieso 2409, P.O. Box 8940577, San Joaquín 8320000, Santiago, Chile
2 Laboratorio de Desarrollo Analítico y Quimiometría (LADAQ), Cátedra de Química Analítica I, Facultad de Bioquímica y Ciencias Biológicas, Universidad Nacional del Litoral, Ciudad Universitaria, Santa Fe 3000, Argentina
3 Consejo Nacional de Investigaciones Científicas y Técnicas (CONICET), Godoy Cruz CP C1425FQB, Buenos Aires 2290, Argentina
4 Department of Materials Science and Engineering, University of Sheffield, Sheffield S1 3JD, UK
5 Instituto de Investigaciones en Físico-Química de Córdoba (INFIQC), Departamento de Físicoquímica, Facultad de Ciencias Químicas, Universidad Nacional de Córdoba, Ciudad Universitaria, Córdoba 5000, Argentina
6 Laboratorio de Fisicoquímica y Electroquímica del Estado Sólido, Facultad de Química y Biología, Universidad de Santiago de Chile, Av. Libertador Bernardo O'Higgins n◦ 3363, Santiago 9160000, Chile
7 Centro de Investigación de Procesos Redox (CiPRex), Universidad de Chile, Sergio Livingstone Polhammer 1007, Independencia 8330015, Santiago, Chile
8 Advanced Center for Chronic Diseases (ACCDiS), Universidad de Chile, Sergio Livingstone Polhammer 1007, Independencia 8330015, Santiago, Chile
* Correspondence: domingo.ruiz@usach.cl (D.R.-L.); sbollo@ciq.uchile.cl (S.B.)

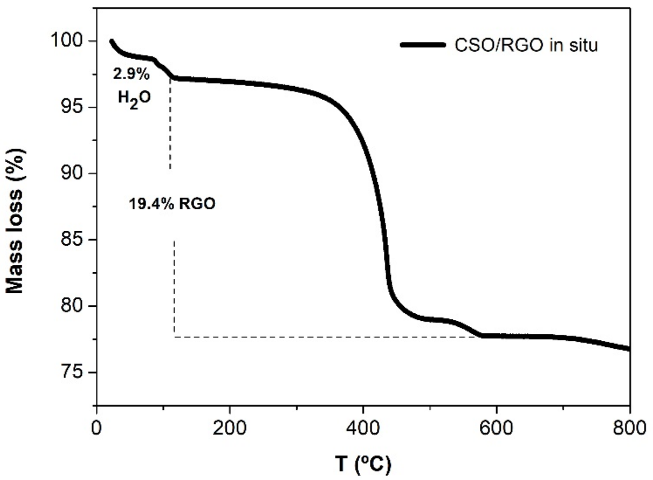


**Figure S1**. TGA analysis for CSO/RGO *in-situ*.

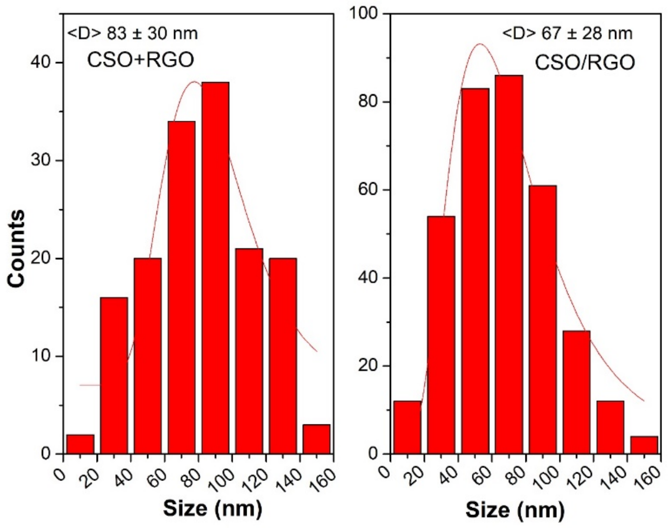


**Figure S2**. Particle histogram distribution.

micromachines

MDPI

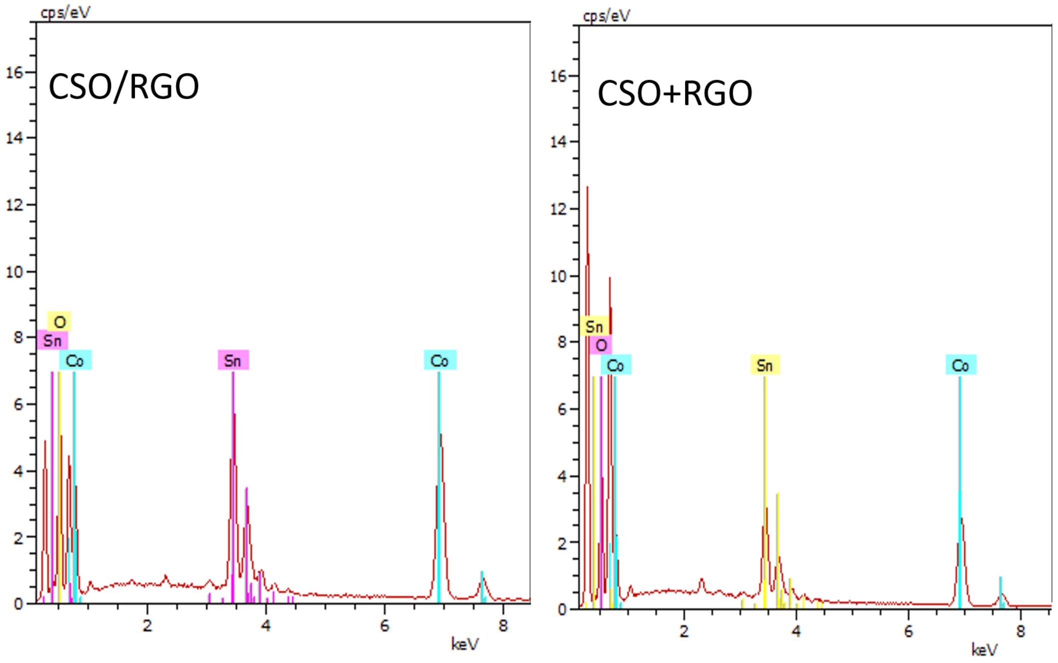


**Figure S3.** EDX spectrum for CSO/RGO and CSO+RGO.

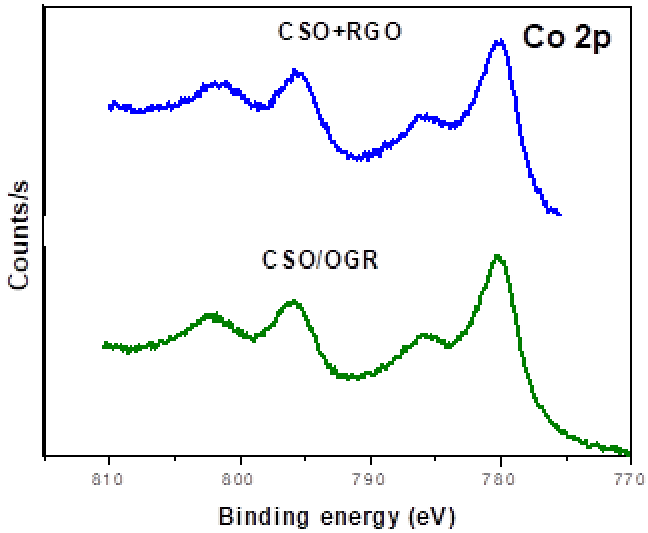


**Figure S4.** Co 2p X-ray photoelectron spectroscopy (XPS) spectra for CSO+RGO and CSO/RGO materials.

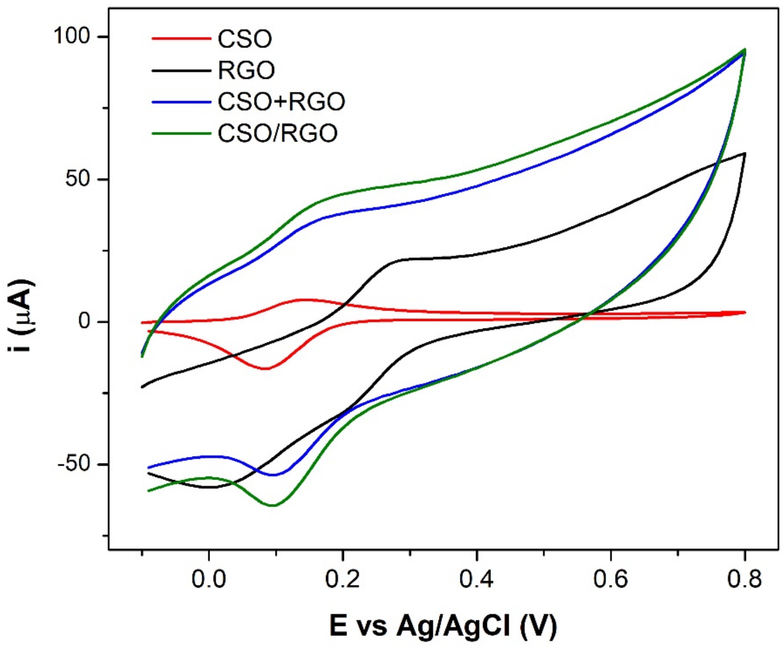


**Figure S5**: Cyclic voltammograms of 0.5 mM ferrocene methanol in PBS pH 7.4 using different modified electrodes.

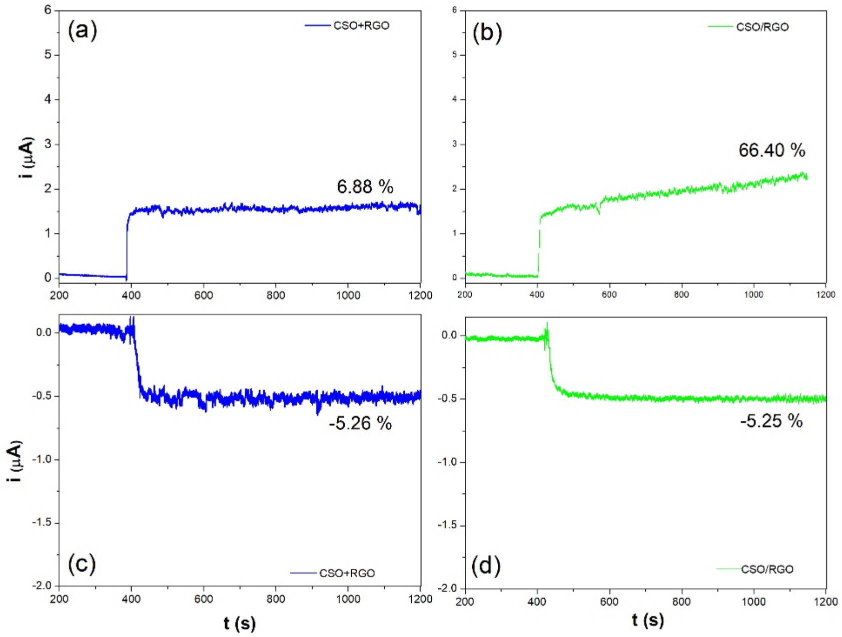


**Figure S6.** Stability of the oxidation response of oxidation of 0.1 mM $H_2O_2$ (a) CSO+RGO and (b) CSO/RGO applying 0.300 V and reduction of 0.1 mM $H_2O_2$ (c) CSO+RGO and (d) CSO/RGO applying −0.400 V.

micromachines

MDPI

**Table S1:** Sensing performance comparison of our systems with other sensors using RGO or metallic oxides.

| Electrode | Electrolyte | Potential (V) | Sensitivity ($\mu$AmM$^{-1}$cm$^{-2}$) | LOD ($\mu$M) | Ref. |
|---|---|---|---|---|---|
| $Co_2TiO_4$/RGO | NaOH (pH12) | −0.400 | 106 | 2.1 | [1] |
| $NiCo_2S_4$/RGO | NaOH (pH13) | −0.450 | 118.5 | 0.19 | [2] |
| $MnO_2$/MWCNT | PBS (pH7.0) | −0.400 | 13.9 | 6.97 | [3] |
| Ni–$Fe_3O_4$@RGO | PBS (pH7.4) | −0.650 | 6 $\mu$AM$^{-1}$ | 0.2 | [4] |
| $Co_2SnO_4$/RGO | NaOH (pH12) | −0.400 | 74 | 7.4 | This work |
| $Co_2SnO_4$+RGO | NaOH (pH12) | −0.400 | 80 | 7.7 | This work |

## References


1. Venegas, C.J.; Gutierrez, F.A.; Eguílaz, M.; Marco, J.F.; Reeves-McLaren, N.; Rivas, G.A.; Ruiz-León, D.; Bollo, S. Co2TiO4/Reduced Graphene Oxide Nanohybrids for Electrochemical Sensing Applications. *Nanomaterials* **2019**, *9*, 1–18. https://doi.org/10.3390/nano9111611.
2. Wang, M.; Ma, J.; Guan, X.; Peng, W.; Fan, X.; Zhang, G.; Zhang, F.; Li, Y. A Novel H2O2 Electrochemical Sensor Based on NiCo2S4 Functionalized Reduced Graphene Oxide. *J. Alloys Compd.* **2019**, *784*, 827–833. https://doi.org/10.1016/j.jallcom.2019.01.043.
3. Dinesh, M.; Revathi, C.; Haldorai, Y.; Rajendra Kumar, R.T. Birnessite MnO2 Decorated MWCNTs Composite as a Nonenzymatic Hydrogen Peroxide Sensor. *Chem. Phys. Lett.* **2019**, *731*, 136612. https://doi.org/10.1016/j.cplett.2019.136612.
4. Kaplan, S.; Suna Karatekin, R.; Kahya Dudukcu, M.; Avcı, G. A Novel Ni–Fe3O4@s-RGO/GCE Electrode for Electrochemical Detection of H2O2. *Mater. Chem. Phys.* **2023**, *294*, 127051. https://doi.org/10.1016/j.matchemphys.2022.127051.
